# Analysis of cervical resistance during continuous controllable balloon dilatation: controlled clinical and experimental study

**DOI:** 10.1186/s13063-015-1003-8

**Published:** 2015-10-28

**Authors:** Petar Arsenijevic, Marko Milosevic, Aleksandar Zivanovic, Biljana Milicic, Branislav Jeremic, Nenad Filipovic, Zoran Protrka, Petar Todorovic, Slobodan Arsenijevic

**Affiliations:** Clinic of Gynaecology and Obstetrics, Clinical Centre Kragujevac, Faculty of Medical Sciences, University of Kragujevac, 69 Svetozara Markovica Street, Kragujevac, 34000 Serbia; Faculty of Engineering, University of Kragujevac, 6 Sestre Janjic Street, 34000 Kragujevac, Serbia; Faculty of Dental Medicine, University of Belgrade, 6 Dr Subotica Street, 11000 Belgrade, Serbia

**Keywords:** Cervical resistance, Continuous controllable balloon dilatation, Measurement and analysis

## Abstract

**Background:**

Hydraulic dilatation is a novel method of cervical dilatation that is based on continuous controllable dilatation (CCBD) by the pumping of fluid into the balloon extension of the system. The main advantage of this procedure is that it allows control of and insight into the process of cervical dilatation.

**Methods:**

For the purposes of our research, we created a new and upgraded system for CCBD which consists of a programmed hydrostatic pump connected to a balloon extension. With regard to our aim to precisely measure and determine the location of the cervical resistance, we placed two pressure-measuring films, one on the top and one on the bottom of the balloon extension. This study included 42 patients in whom cervical resistance was measured before suction curettage.

**Results:**

Cervical dilatation and measurement of cervical resistance were successful in all patients. The analysis of the pressure-measuring films showed that the points of highest resistance were located in the zone of the internal cervical os and that these values were much higher than those in the zone of the external cervical os (0.402 versus 0.264 MPa at the upper pressure-sensitive film; 0.387 versus 0.243 MPa at the lower pressure-sensitive film). This study also showed that an increase in cervical resistance in the zone of the internal cervical os was followed by an increase in cervical resistance in the zone of the external cervical os.

**Conclusions:**

During CCBD, the internal cervical os is the centre of cervical resistance, and the values do not decline with the number of miscarriages or the number of previous births.

**Trial registration number:**

ISRCTN Registry identifier: ISRCTN30949871. Date of registration: 13 May 2015.

**Electronic supplementary material:**

The online version of this article (doi:10.1186/s13063-015-1003-8) contains supplementary material, which is available to authorized users.

## Background

Anatomically, the cervix is part of the uterus, but functionally it is a unique organ. By definition, the cervix represents both a barrier and a connection between the internal female genital organs and the environment [1]. *Cervical dilatation* is a term that refers mostly to the physiological dilatation that occurs during childbirth, although artificial dilatation of the cervical canal is a common procedure in gynaecological practice that is used for both therapeutic and diagnostic procedures, such as hysteroscopy, explorative curettage or placement of intrauterine contraceptive devices [2–4]. The most common method for cervical dilatation is to use Hegar dilators. This method requires significant force, which may lead to permanent damage of the cervical canal [5, 6]. Other methods of cervical dilatation involve the use of osmotic dilators or prostaglandin analogues, which are impractical, often nonfunctional and cause undesirable effects such as cervical haemorrhage or uterine cramping [7–9].

Cervical dilatation by continuous controllable balloon dilatation (CCBD) is a relatively new method that is based on the continuous and controllable pumping of fluid into the balloon extension of the dilatation device [10]. The main advantage of CCBD is that it gives the physician control over the dilatation process and makes the procedure safer for the patient and less stressful for the physician than other methods. In recent studies, CCBD proved to be more reliable and less invasive than other methods and caused significantly less damage to the cervical tissue compared with use of Hegar dilators [11]. CCBD is a safer and more reliable method of cervical dilatation than other methods and can also be used to monitor and analyse the insufficiently researched process of artificial dilatation of the uterine cervix. In our present study, we sought to precisely determine the resistance given by the uterine cervix during dilatation using the CCBD method.

## Methods

### Study design

We conducted a prospective, controlled, clinical and experimental study at the Clinic of Gynaecology and Obstetrics, Clinical Centre, Kragujevac, Serbia. The study was approved by the ethics committee of the Clinical Centre, Kragujevac, Serbia (approval number 01-4169). The authors vouch for the completeness and accuracy of the data and analyses. An independent data and safety monitoring board monitored the study and reviewed the protocol compliance and outcome data.

### Study patients

This research study involved 42 patients who were hospitalised for termination of an unwanted pregnancy in the fertility control ward of the Gynaecology and Obstetrics Clinic in Kragujevac, Serbia, between May 2014 and May 2015. The inclusion criteria were age between 18 and 40 years, pregnancy verified by ultrasound, singleton pregnancy, gestational age of 10 weeks or less, absence of uterine bleeding or cramping, cervix and uterus without pathological changes and closed external cervical os (ECO). If any of the inclusion criteria were not met, the patient was not enrolled in the study. Before each experiment was performed, the dimensions of the cervix (length, anteroposterior and laterolateral diameters) and the location of the ECO and internal cervical os (ICO) were determined by ultrasound. In addition, each patient’s medical history with regard to previous births (by vaginal delivery) and abortions was determined. The experiments were performed with the understanding and appropriate informed consent of each patient (Additional files 1 and 2).

### Study methods

**Fig. 1:**
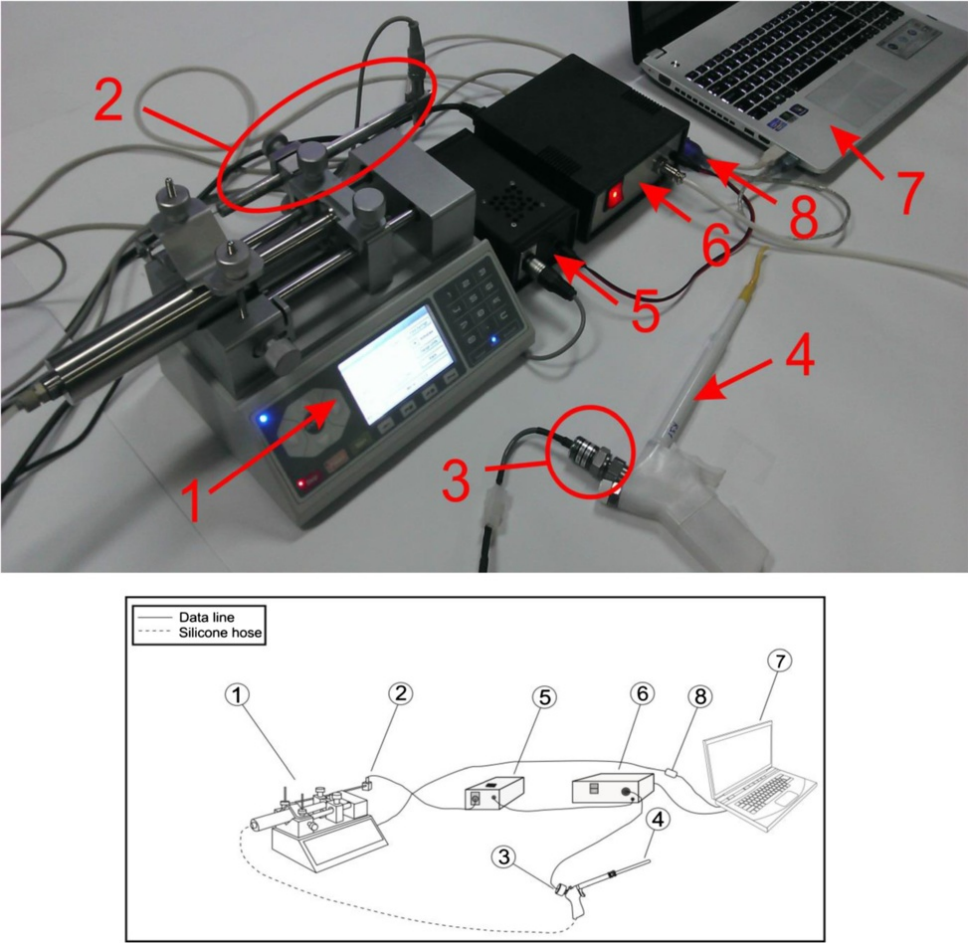
Upgraded system for continuous controllable balloon dilatation: Nexus 6000 hydrostatic pump (National Instruments, Austin, TX, USA) (*1*), linear variable differential transformer displacement sensor (*2*), pressure sensor (*3*), dilatator device (*4*), driver for displacement sensor (*5*), enclosure with USB-6008 data acquisition card (*6*), laptop computer (*7*) and RS232 to USB converter (*8*)

For the purpose of our research, we modified and upgraded the system for CCBD [11]. In the upgraded CCBD system, controllability over the process of dilatation is met on two levels: control over the maximum diameter of dilatation and control of the parameters of the dilatation process (i.e., pressure in the balloon extension, dilatation speed). These two levels of control are reached by using the Nexus 6000 hydrostatic pump (Chemyx, Stafford, TX, USA) and our original software application that allows the dilatation process to operate under strictly controlled conditions (dilatation process lasted 100 seconds; pressure in the balloon extension did not exceed 2 MPa). The tracking and measurement of the dilatation parameters were performed by the pressure sensor, which is located on the dilatator device, as well as by the displacement sensor, which is attached to the hydrostatic pump, as shown in Fig. 1.

**Fig. 2:**
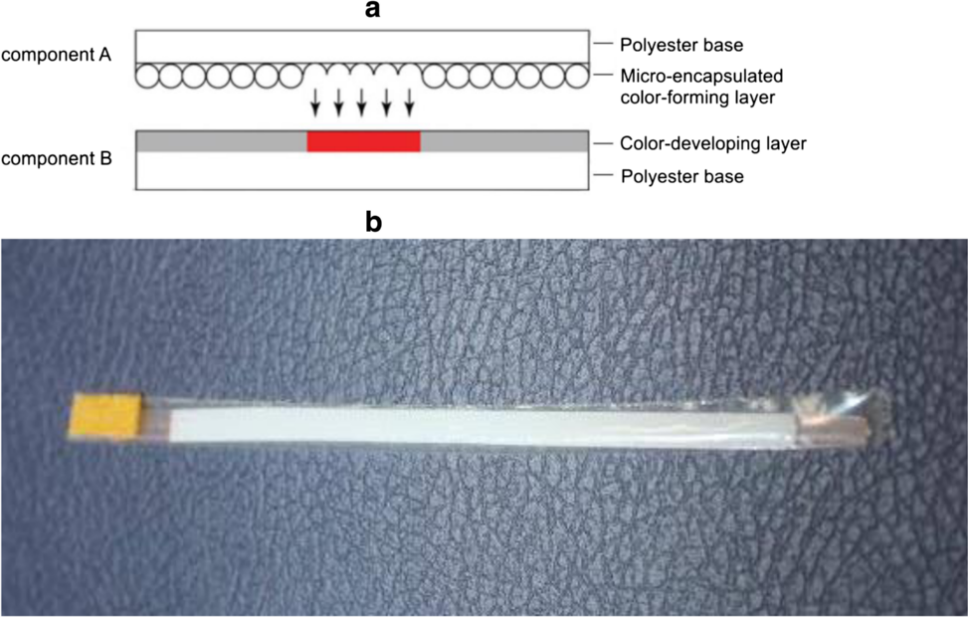
Pressure-sensitive film. **a** Scheme of the pressure sensitive film; **b** Modified pressure sensitive film before dilatation

In our aim to precisely measure and basically map the resistance along the cervical canal during CCBD, we used the following pressure-sensitive films (PSFs): FUJIFILM Ultra Super Low Pressure (Fujifilm, Tokyo, Japan). Under the influence of external force on the PSF, microcapsules in component A of the film rupture and the released red paint is absorbed by the special material in component B, as shown in Fig. 2a. The width of the PSF used in our study was 3 mm and the length was 70 mm. Adhesive tape was placed at both ends of the PSF to keep components A and B together during the experiment. We also placed adhesive tape at the top ends of the PSF to avoid their movement during placement of the balloon extension in the cervical canal, as shown in Fig. 2b. Owing to the presence of cervical mucus and blood during the experiment, we had to isolate our PSF with transparent plastic foil. Finally, in every experiment, we used two PSFs, which were placed on the top and the bottom, respectively, of the balloon extension of the CCBD system.

After completion of dilatation, the PSFs were collected, scanned and converted into their respective digital forms, which were further analysed using the MATLAB program (MathWorks, Natick, MA, USA). The final versions of the processed PSFs are displayed in the Results section.

### Statistical analysis

All of the statistical analyses were performed with IBM SPSS 22.0 for Windows software (IBM, Armonk, NY, USA). All data were numeric. A normal distribution of continuous data was tested using the Kolmogorov–Smirnov test. The correlation between the observed parameters was analysed using Pearson’s (parametric data) and Spearman’s (nonparametric data) correlation coefficients. All of the data in this study are expressed as the mean ± standard error. *p* < 0.05 was considered significant.

## Results

The values of the cervical resistance during CCBD on the upper PSF, which were measured in the zone of the ICO, ranged from 0.144 MPa to 0.559 МРа, with an average of 0.402 ± 0.097 МРа. As for the ECO, the values of the cervical resistance during CCBD on the upper film ranged from 0.043 MPa to 0.467 MPa, with an average of 0.264 ± 0.108 MPa. Further analysis showed that the values of cervical resistance in the zone of the ICO were in positive statistical correlation with the values of the cervical resistance measured in the zone of the ECO (*p* = 0.000). However, no statistically significant correlation was found between the cervical resistance measured in the zone of the ICO and the number of previous births or the number of previous miscarriages, as presented in Tables 1 and 2.

**Table 1:** Correlation of cervical resistance measured in the zone of the ICO on the upper pressure-sensitive film and other parameters

	Upper film ICO	*p* Value
Number of births	ρ = −0.022^a^	0.891
Number of miscarriages	ρ = 0.025^a^	0.878
Number of abortions	ρ = 0.024^a^	0.881
Upper film ECO	*r* = 0.611^b^	0.000^c^
Lower film ICO	*r* = 0.169^b^	0.290
Lower film ECO	*r* = 0.255^b^	0.107

*ICO* internal cervical os, *ECO* external cervical os

^a^Spearman’s correlation coefficient

^b^Pearson’s correlation coefficient

^c^Statistically significant correlation

**Table 2:** Correlation of the cervical resistance values measured in the zone of ECO on the upper pressure-sensitive film and other parameters

	Upper film ECO	*p* Value
Number of births	ρ = 0.009^a^	0.956
Number of miscarriages	ρ = 0.042^a^	0.796
Number of abortions	ρ = 0.034^a^	0.831
Upper film ICO	*r* = 0.611^b^	0.000^c^
Lower film ICO	*r* = 0.207^b^	0.194
Lower film ECO	*r* = 0.657^b^	0.000^c^

*ICO* internal cervical os, *ECO* external cervical os

^a^Spearman’s correlation coefficient

^b^Pearson’s correlation coefficient

^c^Statistically significant correlation

The values for cervical resistance during CCBD on the lower PSF were measured in the zone of the ICO and ranged from 0.135 МРа to 0.582 МРа, with an average of 0.387 ± 0.089 МРа. As for the ECO, the values for cervical resistance on the lower PSF ranged from 0.047 МРа to 0.518 МРа, with an average of 0.243 ± 0.120 МРа. Similar to the upper PSF, a positive statistical correlation was found between the values of cervical resistance measured in the zones of the ICO and ECO (*p* = 0.003), whereas no statistically significant correlation was found for the other parameters that were investigated in this study, as shown in Tables 3 and 4.

**Table 3:** Correlation of the cervical resistance values measured in the zone of the ICO on the lower pressure-sensitive film and other parameters

	Lower film ICO	*p* Value
Number of births	ρ = −0.109^a^	0.497
Number of miscarriages	ρ = −0.083^a^	0.604
Number of abortions	ρ = 0.007^a^	0.966
Lower film ECO	*r* = 0.455^b^	0.003^c^
Upper film ICO	*r* = 0.169^b^	0.290
Upper film ECO	*r* = 0.207^b^	0.194

*ICO* internal cervical os, *ECO* external cervical os

^a^Spearman’s correlation coefficient

^b^Pearson’s correlation coefficient

^c^Statistically significant correlation

**Table 4:** Correlation of the cervical resistance values measured in the zone of ECO on the lower pressure-sensitive film and other parameters

	Lower film ECO	*p* Value
Number of births	ρ = −0.054^a^	0.738
Number of miscarriages	ρ = 0.035^a^	0.829
Number of abortions	ρ = −0.118^a^	0.462
Lower film ICO	*r* = 0.455^b^	0.003^c^
Upper film ICO	*r* = 0.255^b^	0.107
Upper film ECO	*r* = 0.657^b^	0.000^c^

*ICO* internal cervical os, *ECO* external cervical os

^a^Spearman’s correlation coefficient

^b^Pearson’s correlation coefficient

^c^Statistically significant correlation

**Fig. 3:**
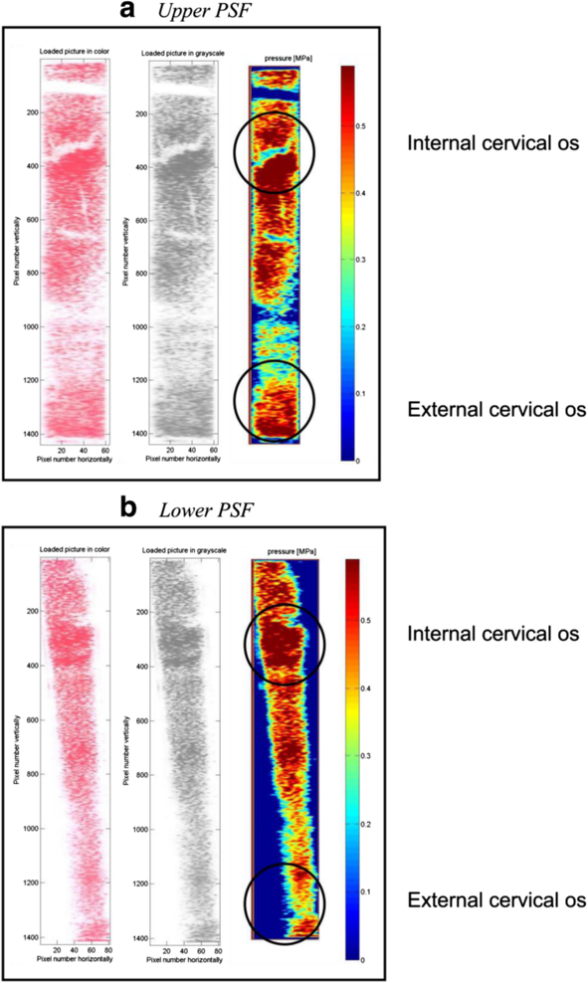
Pressure-sensitive films (PSFs) of patient 3 with cervical diameters and marked zones of internal and external cervical os: cervical length 54 mm, anteroposterior diameter 26 mm and laterolateral diameter 27 mm. **a** Upper PSF **b** Lower PSF

**Fig. 4:**
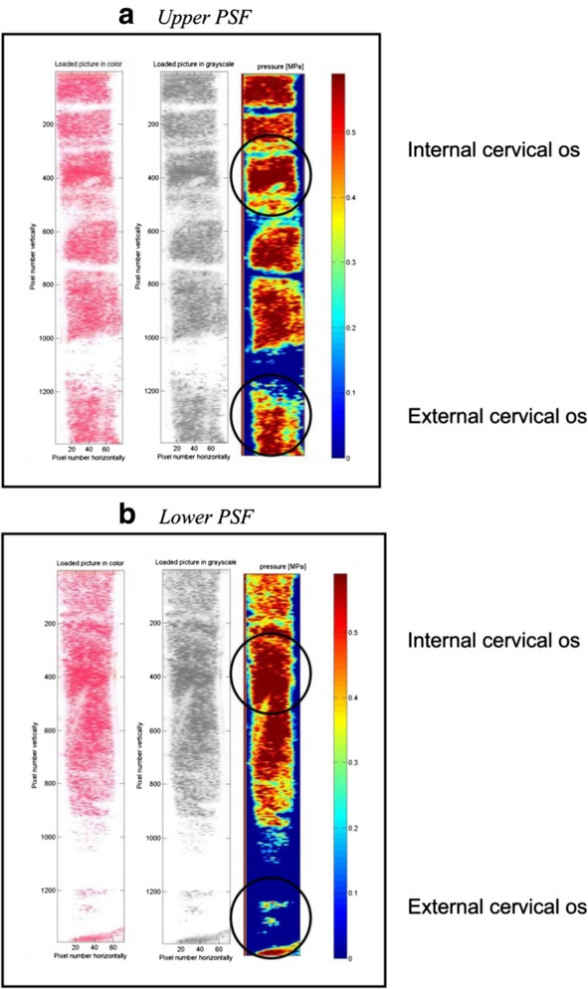
Pressure-sensitive films (PSFs) of patient 36 with cervical diameters and marked zones of internal and external cervical os: cervical length 50 mm, anteroposterior diameter 28 mm and laterolateral diameter 30 mm. **a** Upper PSF **b** Lower PSF

Processed PSFs gave us insight into the map of cervical resistance during dilatation with the CCBD system. As shown in Figs. 3 and 4, the zones of the highest resistance are matched with the anatomic sites of the ICO and ECO of the cervix, predicted by cervical dimensions acquired by ultrasound, before the intervention.

## Discussion

The use of the CCBD system allowed us to investigate, for the first time to our knowledge, the phenomenon of cervical dilatation during the process itself. Previous research on the mechanical properties of cervical tissue is scarce, and the research that has been conducted is mostly limited to studies in ex vivo conditions. One of the first studies on the mechanical properties of the human cervix was conducted by Conrad et al., who used human cervical tissue samples that were obtained after hysterectomy. Their research showed that tissue samples from the ICO exhibited the greatest resistance to the uniaxial tension [12, 13]. Later, Petersen et al. conducted research on cervical tissue samples of nonpregnant women. They showed that the biomechanical strength of the uterine cervix is passive and that it is derived from the high concentration of collagen fibres in the cervical stroma [14–16]. One of the rare in vivo studies on the mechanical characteristics of cervical tissue was conducted by Mazza et al. Eight experiments were performed in vivo, and four were performed ex vivo. The results showed that the ex vivo mechanical response of the cervical tissue did not differ considerably from that observed in vivo [17, 18].

The research study most similar to ours was conducted by Kiwi et al. They used the specially designed balloon extensions to dilate the cervix and showed that the elastic properties of the cervix decline as the number of previous miscarriages increases [19, 20]. The same conclusion was reached by Anthony et al., who measured the cervical resistance index in nonpregnant women before some of the routine gynaecological interventions were performed. The results of their study showed that women with a history of one or more miscarriages have much lower values of cervical resistance than those without previous miscarriages [21, 22].

The main aim of our research was to precisely measure, analyse and create a map along the cervical canal during the dilatation of the uterine cervix. With the use of the CCBD system and the application of the PSFs, we managed to locate every point of resistance along the upper and lower sides of the cervical canal. An analysis of the PSFs showed that the points of greatest resistance are in the zone of the ICO and that these values are much higher than those in the zone of the ECO for both PSFs. The reason for this finding derives from the difference between the two cervical ossa. With the exception of densely packed collagen fibres, the ICO contains a high concentration of arranged muscle fibres. However, a statistically positive correlation exists between the values of cervical resistance measured in the zones of the ICO and ECO for both of the PSFs. That means that despite the fact that the ICO is the centre of cervical resistance during CCBD, the dilatation occurs only after simultaneous loosening of both cervical ossa. These findings are in accordance with those of other studies of the mechanical properties of the uterine cervix. On the contrary, however, in our study, no statistically significant correlation was found between values of cervical resistance and the number of previous miscarriages. This result is opposite to that of previous studies. The reason for this contradiction might lie in the fact that both the cervical resistance index and the elastic properties of the cervix were previously investigated in nonpregnant women, whereas our study on cervical resistance was performed in women before the termination of unwanted pregnancies, before 10 weeks of gestation.

## Conclusions

During CCBD, the ICO is the centre of cervical resistance, and the values of the measured resistance are in positive correlation with the resistance measured in the zone of the ECO. There is no significant correlation between cervical resistance during dilatation with CCBD system and number of previous births or miscarriages. Future investigation of cervical resistance and response to CCBD requires a collaborative effort between engineers and clinicians and will provide further insight into the biomechanical aspects of the cervical tissue.

## Additional files

Additional file 1:
**Consolidated Standards of Reporting Trials (CONSORT) 2010 checklist.** (DOC 218 kb) Additional file 2:
**CONSORT 2010 flow diagram.** (DOC 52 kb)

## Abbreviations

CCBD: Continuous controllable balloon dilatation
ECO: External cervical os
ICO: Internal cervical os
PSF: Pressure-sensitive film
