# Supplementary material for: Analysis of cervical resistance during continuous controllable balloon dilatation: controlled clinical and experimental study
Source: Trials. 2015 Oct 28;16:485. doi: 10.1186/s13063-015-1003-8 (PMC4625528; doi:10.1186/s13063-015-1003-8)
Supplement: Additional file 2: — CONSORT 2010 flow diagram. (DOC 52 kb) [file 13063_2015_1003_MOESM2_ESM.doc]

**
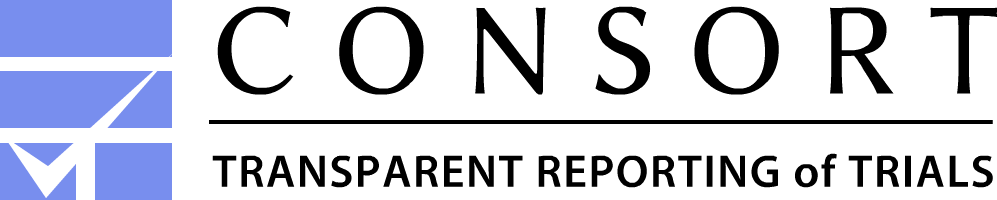
**

**CONSORT 2010 Flow Diagram**

**Allocation**

**Analysis**

**Follow-Up**

**Enrollment**

Assessed for eligibility (n=83 )

Excluded (n= 27 )

♦  Not meeting inclusion criteria (n=24 )

♦  Declined to participate (n=3 )

♦  Other reasons (n=0 )

Analysed (n= 42 )
♦ Excluded from analysis (give reasons) (n= 0 )

Lost to follow-up (give reasons) (n=8 ) problems with the pressure sensitive films

Discontinued intervention (give reasons) (n=1 ) technical difficulties with the CCBD system

Allocated to intervention (n= 56 )

♦ Received allocated intervention (n= 51 )

♦ Did not receive allocated intervention (give reasons) (n=5 ); didn’t want to terminate the pregnancy

Lost to follow-up (give reasons) (n= 8 )

Discontinued intervention (give reasons) (n=1 )

Allocated to intervention (n=56 )

♦ Received allocated intervention (n= 51)

♦ Did not receive allocated intervention (give reasons) (n=5 )

Analysed (n=42 )
♦ Excluded from analysis (give reasons) (n=0 )

Randomized (n=0 )
